# Rise of the killer plants: investigating the antimicrobial activity of Australian plants to enhance biofilter-mediated pathogen removal

**DOI:** 10.1186/s13036-019-0175-2

**Published:** 2019-06-06

**Authors:** P. Galbraith, R. Henry, D. T. McCarthy

**Affiliations:** Environmental and Public Health Microbiology Laboratory (EPHM Lab), Monash Water for Liveability, Department of Civil Engineering, Monash University, Wellington Road, Clayton, Victoria 3800 Australia

**Keywords:** Antimicrobial, Vegetation, Urban stormwater, Biofilter, Fecal microorganisms, Pathogens, WSUD

## Abstract

**Background:**

Biofilters are soil-plant based passive stormwater treatment systems which demonstrate promising, although inconsistent, removal of faecal microorganisms. Antimicrobial-producing plants represent a safe, inexpensive yet under-researched biofilter design component that may enhance treatment reliability. The mechanisms underlying plant-mediated microbial removal in biofilters have not been fully elucidated, particularly with respect to antimicrobial production. The aim of this study was therefore to inform biofilter vegetation selection guidelines for optimal pathogen treatment by conducting antimicrobial screening of biofilter-suitable plant species. This involved: (1) selecting native plants suitable for biofilters (17 species) in a Victorian context (southeast Australia); and (2) conducting antimicrobial susceptibility testing of selected plant methanolic extracts (≥ 5 biological replicates/species; 86 total) against reference stormwater faecal bacteria (*Salmonella enterica subsp. enterica* ser. Typhimurium*, Enterococcus faecalis* and *Escherichia coli*).

**Results:**

The present study represents the first report on the inhibitory activity of polar alcoholic extracts from multiple tested species. Extracts of plants in the Myrtaceae family, reputed for their production of antimicrobial oils, demonstrated significantly lower minimum inhibitory concentrations (MICs) than non-myrtaceous candidates (*p* < 0.0001). *Melaleuca fulgens* (median MIC: 8 mg/mL; range: [4–16 mg/mL])*, Callistemon viminalis* (16 mg/mL, [2–16 mg/mL]) and *Leptospermum lanigerum* (8 mg/mL, [4–16 mg/mL]) exhibited the strongest inhibitory activity against the selected bacteria (*p* < 0.05 compared to each tested non-myrtaceous candidate). In contrast, the Australian biofilter gold standard *Carex appressa* demonstrated eight-fold lower activity than the highest performer *M. fulgens* (64 mg/mL, [32–64 mg/mL]).

**Conclusion:**

Our results suggest that myrtaceous plants, particularly *M. fulgens*, may be more effective than the current vegetation gold standard in mediating antibiosis and thus improving pathogen treatment within biofilters. Further investigation of these plants in biofilter contexts is recommended to refine biofilter vegetation selection guidelines.

**Electronic supplementary material:**

The online version of this article (10.1186/s13036-019-0175-2) contains supplementary material, which is available to authorized users.

## Background

Freshwater scarcity represents a cumulative environmental and humanitarian challenge [1]. With increasing pressures on natural water resources from irresponsible waste disposal, changing land use patterns, climate change and population growth [2], stormwater has gained recognition as an important alternative freshwater resource. Prior to reuse, stormwater must undergo treatment to remove pollutants of concern to human health. The most significant of these are disease-causing faecal microorganisms (faecal pathogens) [3].

A diverse range of faecal pathogens originating from varied sources, including viruses, bacteria and protozoa, can be present in stormwater. Limited studies suggest that pathogen concentrations in stormwater are highly variable, ranging from 8.0 × 10^− 2^ to 1.9 × 10^6^ organisms per litre depending on specific microbial (species, physiology) and environmental (land use, climate and hydrology) factors [4–6]**.** Some pathogens may be present in stormwater at sufficient concentrations to cause disease, particularly those with low infectious doses. For example, *E. coli* O157:H7 (notable haemorrhagic colitis-associated enteropathogen; infectious dose of 1–10 organisms [7]), has been detected in stormwater at levels posing significant disease risk to exposed individuals (up to 1.3 × 10^6^ organisms per litre) [8]. Removal of faecal microorganisms from stormwater is therefore a priority to alleviate health risks associated with downstream harvesting and recreational use.

A range of water sensitive urban design (WSUD) technologies have emerged to facilitate stormwater pollutant removal under a holistic water conservation approach; among these are stormwater biofilters [9]. Biofilters are low-cost, soil-plant based WSUD stormwater treatment systems that demonstrate effective removal of multiple pollutants [10]. When designed according to best practise, these systems can achieve effective reductions in suspended solids (> 95% removal), nitrogen (> 50%), phosphorous (> 65%) and heavy metals (except for Al; > 90%) from stormwater [10–12]. Biofilters have moreover demonstrated promising reductions in faecal microorganism concentrations in stormwater. Three- to four-log removal rates of the common faecal indicator organism (FIO) *E. coli* have been reported in biofilters of varying design [13–16]. Nevertheless, significant differences in removal performance have also been reported in literature [14, 16–18], with certain operational conditions causing removal rates to decline from > 90% to net export within the same system [19, 20].

Microorganism removal performance can decline when intervals between rainfall periods are too short (i.e. back-to-back storm events) or too long (i.e. > 2 weeks), particularly when influent contains high microbial loads [18]. Performance also varies over biofilter lifespan owing to changes in microbial retention (straining and adsorption) with ongoing hydraulic compaction and sediment accumulation on the biofilter surface [14]. Due to their ongoing variability in removal performance, biofilters do not consistently meet removal targets for stormwater recycling (other than some irrigation purposes) [5, 16]. Consequently, additional disinfection steps are generally required prior to reuse of biofilter-treated stormwater (e.g. chlorination, UV irradiation). Removal inconsistency during the course of biofilter operation represents an impediment to the uptake of these systems by urban water managers [21].

To seek opportunities for improving pathogen removal consistency, research has focused on biofilter design. The majority of this work has been conducted on enhancing microbial retention within biofilters, such as through inclusion of a saturated/submerged zone (SZ) [22] and adsorption-promoting filter media [23–25]. More recently, researchers have attempted to enhance in-situ pathogen inactivation processes through the addition of antimicrobial filter media [21, 26–28]. Results have demonstrated that some configurations were capable of achieving consistent 2-log reductions in *E. coli* within the first 40 min of infiltration [26]. While antimicrobial media offer an excellent opportunity for improved pathogen inactivation, many require further optimisation to decrease costs, remove true pathogens (not solely FIOs), maintain performance during operation (especially with cold temperatures and clogged conditions) [21], or resolve leaching of antimicrobial amendments into effluent [26, 28]. To complement research into advancing filter media for microbial removal, other biofilter design elements are undergoing further investigation.

One crucial biofilter design feature affecting pathogen removal is vegetation. Plants represent a safe, inexpensive and easily adaptable biofilter component with established efficacy in facilitating the removal of multiple stormwater pollutants, particularly nutrients and metals [29]. Plants directly assimilate dissolved pollutants, while their roots provide solid surfaces on which biofilms form and adsorb colloidal and suspended sediment-bound pollutants [30, 31]. Preliminary research indicates that plants also play a significant role in faecal microorganism removal within biofilters [22]. For example, systems vegetated with plants possessing extensive root systems have demonstrated the greatest *E. coli* removal from stormwater, potentially due to increased root surface area corresponding with enhanced microbial retention [22, 32, 33]. Plants may also modulate physicochemical parameters within biofilters including UV penetration, soil pH, and concentrations of nutrients, oxygen and carbon dioxide [34–36] depending on plant life stage, genetics and various biotic and abiotic stresses. Although the role of plants in pollutant removal within biofilters has been well-established, the mechanisms by which they govern pathogen removal remain poorly characterised.

Perhaps most importantly, some plants possess antimicrobial properties which may aid in pathogen removal within biofilters. Previous studies have noted that multiple plant species employed in WSUD systems produce antimicrobial compounds [37–41]. In particular, native Australian plants represent a trove of hardy, antimicrobial-producing candidates that may significantly improve pathogen die-off in biofilters. Two Australian plants known for their antimicrobial properties, *Melaleuca incana* and *Leptospermum continentale*, were observed to achieve 10-fold higher *E. coli* reductions in biofilters than other planted systems with similar retention (infiltration) rates [22]. It was hypothesised that the introduction of antimicrobial compounds by these plants augmented pathogen die-off in these systems [22, 42] via litterfall and/or root secretions [42, 43]. A subsequent *E. coli* survival test conducted on root exudate solutions from harvested plants confirmed that *L. continentale* exhibited higher antimicrobial activity than *Carex appressa* [42]. This finding was reinforced by Shirdashtzadeh et al. [43], who conducted antimicrobial screening of seedlings (extracts) and seeds (exudates and extracts) from nine biofilter-suitable plants against *E. coli. Melaleuca ericifolia*, a close relative of *M. incana*, demonstrated the greatest observed inhibitory activity of tested species. In combination, these findings suggest that incorporating highly antimicrobial plants into plant selection guidelines for biofilters may enhance pathogen die-off within these systems.

The leaves, seeds and flowers of a variety of native Australian plants employed in biofilters, including *Allocasuarina* sp., *Acacia* sp., *Melaleuca* sp. and *Leptospermum* sp., are known to contain antimicrobial compounds such as terpenes, triketones and phenols [44–47]. Tissue extracts from these and other Australian native plants often demonstrate strong antimicrobial activity against faecal pathogens [44, 45, 48–50]. It is predicted that a proportion of plant antimicrobials deposited into biofilters originates from litterfall. Antimicrobial compounds may be released from decomposing litter into the top media layers of biofilters, where most faecal bacteria are retained [51, 52]. Antimicrobial compounds in litterfall may interfere with microbial nutritional processes (e.g. decomposition, mineralisation, and humification) and survival [53], potentially resulting in increased die-off of exposed microorganisms at the biofilter surface (via litter accumulation) and subsurface (via aqueous leachates and litter bioturbation). Antimicrobial quality and quantity in litterfall can vary depending on intrinsic plant factors (e.g. species/genotype, age, health, biomass), environmental physicochemical factors (e.g. UV intensity, altitude, temperature, moisture availability, salinity, nutrients, pH), and the soil microbial population (abundance of plant pathogens and commensals/mutualists) [54–59]**.** Litter deposition rates are also influenced by prevailing environmental conditions, with many south-eastern Australian plants depositing larger litter volumes in summer [60]. In this way, environmental modulation of antimicrobial deposition via litterfall may affect pathogen treatment within biofilters.

In addition to litter inputs, plants may release antimicrobial compounds into biofilters via root exudates. Some native biofilter-suitable *Acacia* and *Callistemon* species are known to produce root exudates containing inhibitory compounds [61–63]. For example, Leptospermone, a β-triketone with broad-spectrum antibacterial [64] and allelopathic activity, is produced by *Callistemon sp.* (Myrtaceae) roots to suppress weeds around the plant’s base [63]. Prosser et al. [65] suggested that rhizodeposition of this and other antimicrobial compounds potentially enhanced the observed removal of *E. coli* in soil vegetated with related species (*Leptospermum* and *Kunzea* sp., family Myrtaceae). Furthermore, Chandrasena et al. [22] suggested that antimicrobial root exudates contributed to improved pathogen removal in biofilters planted with myrtaceous *Leptospermum* and *Melaleuca* species. Nevertheless, the influence of root exudates on biofilter-mediated pathogen removal remains uncharacterised.

Antimicrobial vegetation represents an under-investigated biofilter design feature which may have significant pathogen inactivation potential [22]. Some independent antimicrobial susceptibility testing (AST) assays have been conducted on the exudates and tissue extracts of certain plant species that have been employed in biofilters [44–47]. However, only a single study to date has conducted antimicrobial testing on plants selected for their specific suitability in biofilters (seed exudates, seed and seedling extracts) against a single stormwater faecal microorganism (*E. coli*) [43]. The antimicrobial effect of mature plants selected for their specific suitability in biofilters against stormwater pathogens remains yet to be investigated. Furthermore, the antimicrobial effect of leaf litter in biofilters remains uncharacterised, despite being a potential facilitator of pathogen treatment in these systems.

The aim of this study was therefore to conduct antimicrobial screening of suitable mature Australian plants to inform biofilter vegetation selection guidelines for optimal pathogen removal. The following objectives were applied to meet this aim: (1) selecting appropriate native plant candidates for survival and function in biofilters in a Victorian context (south-eastern Australia); and (2) conducting antimicrobial susceptibility testing (AST) of selected plants against multiple reference stormwater faecal bacteria (*Salmonella enterica subsp. enterica* ser. Typhimurium*, Enterococcus faecalis* and *Escherichia coli*). This study elucidates several antimicrobial plant species with specific suitability in biofilters that demonstrate noteworthy potential for enhancing pathogen treatment in these systems. The findings of this study represent an important contribution to the development of best-practise vegetation selection guidelines, and underpin prospective research into the role of vegetation in microorganism removal within WSUD systems.

## Methods

### Plant selection

Plant species from sedge, shrub and tree retail stock lists of nine major native plant nurseries in Melbourne (Victoria, Australia) were combined for preliminary screening. Selection from native nurseries ensured that candidates fulfilled the following criteria: 1) native to Australia; 2) easily accessible to stakeholders for purchase and implementation in biofilters; and 3) unlikely to become invasive weeds once established. Native Australian plants were preferentially examined owing to many possessing adaptations that confer inherent suitability to biofilter environments (e.g. resistance to drought, high temperatures, high UV intensity, poorly structured soils with low organic matter/limited nutrients, and in some cases, heavy metal exposure) [66, 67]. Each species in the combined list of plants was manually ranked with consideration to multiple weighted criteria (Table 1). These criteria included adaptation to common biofilter conditions (i.e. drought-tolerance, ability to grow in sandy soil, ability to withstand periodic temporary inundation), possession of deep, extensive root systems (previously associated with effective removal of pathogens and other pollutants [22, 29, 68], lack of nitrogen fixing capability (linked to poor N removal [69]), high growth rate (associated with optimal nutrient removal [29, 70]), and suitable sizing for integration into most streetscape biofilter systems (< 10 m in height) (Table 1). Plant species were subsequently assigned an “antimicrobial score” based on literature searches to provide an indication of known antimicrobial activity (Additional file 1: Table S1 description). Species demonstrating very low (< 1) or high (> 30) antimicrobial scores were considered for selection as “putatively antimicrobial” and “putatively non-antimicrobial” test plants, respectively. Taking all the above criteria and their relative weightings into account, species ranks (0–5) were assigned to each plant. Detailed steps on calculating species ranks are outlined in Additional file 1: Table S1 footnotes.

**Table 1:** Weighted criteria for selecting test plant species based on suitability in field-scale biofilters and known antimicrobial production

Selection criteria	Description	Reference	Weighting of importance
Primary considerations for species selection			
Availability in nurseries	Available from ≥1 of 9 major native nurseries in Melbourne.	[29]	✓✓✓✓✓
			Accessible to stakeholders for purchase
Adaptation to biofilter conditions	Species were scored (1–3) based on their ability to maintain healthy growth under south-eastern Australian biofilter conditions (i.e. survival in sandy soil with temporary inundation and extended hot, dry periods). Plants with scores of ≥2 were considered for selection.	[11, 68]	✓✓✓✓✓
			Plants must be adapted to harsh conditions in biofilters for effective performance
Antimicrobial activity of plants	Species were assigned an antimicrobial score based on the number of positive Google Scholar search results associating plant genus with antimicrobial-associated terms*. Species with an antimicrobial score of > 30 and < 1 were considered for selection.	[43]	✓✓✓✓✓
			Parameter essential to answer key objectives of study
Extensive root system	Species were scored (1, 2 or 3) based on root structure characteristics, with 3 representing “very good” (deep, dense, extensive, fine roots), 2 representing “average” and 3 representing “poor” roots (shallow, thick, minimal root systems). Candidates with scores ≥2 were considered for selection.	[22, 29, 68]	✓✓✓✓✓
			Extensive root systems correspond with high pollutant and faecal microorganism removal
Invasive species	Species deemed to have a high risk of becoming invasive, even if native to Australia, were excluded from selection.	[29]	✓✓✓✓✓
			Necessary to avoid ecological damage to surrounding ecosystems in field applications
Plant size	Species typically growing ≤10 m in height and ≥ 1 m in canopy diameter (sedges excepted) were considered for selection. Tall trees are generally impractical or unpopular in streetscape biofilters, while slender shrubs/trees with sparse above-ground biomass have diminished treatment capacity [71] due to their generally lower litterfall and weaker root systems [68].	[29, 68]	✓✓✓✓✓
			Size constraints necessary for successful field application
Secondary considerations for species selection			
Woody plants (shrubs and trees)	Woody species were preferentially selected. Compared with herbaceous species, woody species tend to live longer, root more extensively, grow taller and produce more biomass and leaf litter for improved treatment capacity [72, 73].	[68, 72, 73]	✓✓✓
			Woody plants have associations with multiple criteria that improve suitability and performance in biofilters
Indigenous to Melbourne	Species indigenous to Melbourne were preferentially selected over other Australian natives. Indigenous species are likely to have superior survival rates and provide greater ecological benefits over non-native species. Indigenous plants are also less likely to become invasive or cause environmental harm.	[29, 68]	✓✓✓
			Plants indigenous to Melbourne are preferred, although other Australian natives are suitable depending on biofilter location and treatment context
High past success in biofilters	Species with high past performance in biofilters were preferentially selected.	[11, 29, 68]	✓✓✓
			Multiple pollutant removal for enhanced field application performance
High growth rate	Species with high growth rates were preferentially selected due to associations with improved nutrient removal.	[29, 68]	✓✓
			Multiple pollutant removal for enhanced field application performance
Nitrogen fixation	Species lacking nitrogen-fixing root systems were preferentially selected to avoid compromised nitrogen removal.	[29, 70]	✓✓
			Multiple pollutant removal for enhanced field application performance
Lifespan	Plants with lifespans > 20 years were preferentially selected over shorter-lived species requiring frequent replacement.	[74]	✓✓
			Reduced maintenance costs and disturbance to biofilter function

The weighted/relative importance of each plant selection criterion (right-most column) was denoted by a number of ticks (✓), with five ticks indicating “very high importance”, four ticks “high importance”, three ticks “moderate importance” and two ticks “relatively low importance”

*Details on antimicrobial score assignment are outlined in Table S.1 description. Secondary metabolite publications were incorporated in the overall antimicrobial score for each plant species, owing to secondary metabolite production providing an indication of antimicrobial activity in plants where antimicrobial testing has not yet been conducted [75]. A Spearman correlation rank of 0.86 (*p* < 0.0001) indicates there is a strong correlation between “Antimicrobial” and “Secondary metabolite” Google Scholar publication count for each filtered species (as determined by GraphPad Prism version 7, GraphPad Software, USA)

### Sample collection

Fresh leaf samples were collected from healthy, mature individuals (aged ~ 3 - ~ 70 years old) of species with overall ranks of ≥3 around metropolitan greater Melbourne (29/09/16–17/11/16, spring). Sampling was conducted in botanical gardens, parkland, nature strips and nature reserves of 30 geographically distinct areas, ensuring accurate statistical representation of antimicrobial production variation with exposure to different biotic and abiotic conditions. Species identities of samples were confirmed through expert opinion (horticulturalists, botanists and botanical garden curators) in conjunction with the use of taxonomic keys [76, 77]. Healthy leaves from multiple locations of the canopy (~ 5 g) were collected from each individual to ensure representative foliage sampling. Samples were transported on ice to the laboratory within 5 h of collection and stored at − 80 °C prior to processing.

### Sample processing and extract preparation

Methanolic extracts were prepared from leaf samples for species with > 5 individuals sampled. The method of Wright et al. [78] was applied, with modification. Briefly, leaf samples were thawed, ground coarsely and freeze-dried over 72 h. Subsamples (0.5–2.5 g) of each dried sample were crushed finely and extracted in 50 mL pure methanol (EMSURE® ACS grade, Merck Millipore, Germany) with gentle rotation at 60–80 rpm over 24 h at 25 ± 2 °C. The extract was centrifuged at 3153 rpm (2000 *x g*) for 5 min and the supernatant collected [45]. Aliquots (25 mL in two separate volumes) of each methanolic extract were open-air evaporated over 24 h under a fume hood [79]. Tubes were then weighed to determine extraction efficiency and stored at − 80 °C. The extracted plant material was subsequently resuspended and homogenised in 1% DMSO (Sigma-Aldrich, USA) in deionised water (0.4–1.2 mL volumes) to achieve final concentrations of > 128 mg/mL (at least double 64 mg/mL, the starting concentration for subsequent antimicrobial testing). All resuspended extracts were transferred into new storage vessels, with the final concentrations (mg/mL) calculated by subtracting the weight of any fine material remaining in the original vessel. Resuspended extracts were stored at − 80 °C prior to further processing.

### Reference strains and standardisation of inoculum

*Salmonella enterica subsp. enterica* ser. Typhimurium TM11 (SARA12) (Gram-negative faecal pathogen; abbreviated to *Salmonella* ser. Typhimurium hereafter) [80], *Escherichia coli* ATCC 11775 (Gram-negative FIO) [81, 82] and *Enterococcus faecalis* ATCC 29212 (Gram-positive FIO) [83] were selected as the test organisms for all antimicrobial susceptibility testing (AST) assays. Each reference strain was subcultured by streaking onto Brain Heart Infusion (BHI) agar (Oxoid, UK) from stock solution stored at − 80 °C. BHI plates were incubated at 37 °C for 18 ± 2 h. Inoculum standardisation for each organism was carried out using the colony suspension method to ensure reproducible AST results [83]. Briefly, 3–5 colonies were selected from BHI plates and inoculated into BHI broth. Multiple dilutions of the culture were prepared with optical densities (OD) ranging from 0.01–0.13 at 625 nm [83] as determined by spectrophotometry (DR 5000, Hach spectrophotometer) [84] and quantified (colony forming units/mL i.e. CFU/mL) by standard colony counting procedures. Calibration curves were plotted (cell concentration vs. OD at each dilution) to determine the absorbance at which a cell density of 5 ± 3 × 10^5^ CFU/mL could be achieved, as per CLSI [83] and Sutton [84].

### Antimicrobial susceptibility testing of methanolic plant extracts.

Antimicrobial susceptibility testing of methanolic plant extracts (*n* = 86, 17 species) was conducted following the standardised Clinical Laboratory Standard Institute (CLSI) broth microdilution broth assay [83]. Similarly to Kurekci et al. [45], plant extracts were thawed and examined for microbial contamination by streaking onto BHI and incubating at 35 °C over 24 h. Extracts were centrifuged briefly at 3153 rpm (2000 *x g*) to remove strongly coloured particulates from suspension. The supernatant was diluted in sterile Mueller-Hinton broth (MHB) (Oxoid, UK) to a starting concentration of 64 mg/mL in the initial wells of a 96-well microplate (clear flat bottom TC-treated; Falcon, USA). Two-fold serial dilutions of the initial 64 mg/mL wells were then prepared in horizontal wells (left to right) with MHB, down to a final concentration of 125 μg/mL (final well volumes 100 μL). All samples were prepared in at least duplicate for each test concentration.

*Salmonella* ser. Typhimurium*, E. coli* and *E. faecalis* BHI broth cultures were prepared as described above (see *Reference strains and standardisation of inoculum*) to a final inoculum concentration of 5 × 10^5^ CFU/mL. All wells were inoculated with 5 μL of test organism (~ 2.5 × 10^3^ CFU per well). Inocula concentrations were confirmed by viable plate counts on BHI agar (appropriate range: 5 ± 3 × 10^5^ CFU/mL). Wells containing a negative antimicrobial control (MHB containing 1% DMSO, i.e. the concentration of DMSO in extracts) and a positive antimicrobial control (MHB containing 50 μg/mL gentamicin, Sigma-Aldrich, USA) were prepared in duplicate for comparison. Microplates were sealed and incubated at 35 °C for 18 h under aerobic conditions. To indicate microbial respiratory activity, the colorimetric growth indicator INT (2-p-iodophenyl-3-p-nitrophenyl-5-phenyl tetrazolium chloride, Sigma-Aldrich, USA) dissolved in water (2 mg/mL) was added to each well in 10 μL volumes before incubating microplates for a further 30 min in the dark [85, 86]. Active bacterial growth was indicated by a visible change from colourless to purple-pink, based on the reduction of INT (colourless) to INT-formazan (purple-pink). The lowest extract concentration demonstrating the absence of a colour change was taken as the minimum inhibitory concentration (MIC) [85, 86].

### Statistics

Statistical comparisons were conducted using uncorrected Mann-Whitney (MW) tests (nonparametric 2-group comparisons) and Dunn’s corrected Kruskal-Wallis (DKW) tests (nonparametric > 2 group comparisons). A DKW test was applied to compare each individual plant species (combined test organism MIC data), and comparisons that were significant (< 0.05; *n* = 40 comparisons) were confirmed by post hoc Bonferroni-corrected Mann-Whitney (PBMW) tests [87, 88]. For all significant DKW tests, PBMW tests generated identical outcomes of significance at an alpha level of 0.05. Spearman correlation tests were applied for correlation analyses. All statistical analyses were performed using GraphPad Prism version 7 (GraphPad Software, USA).

## Results and discussion

### Classification of plants

**Fig. 1:**
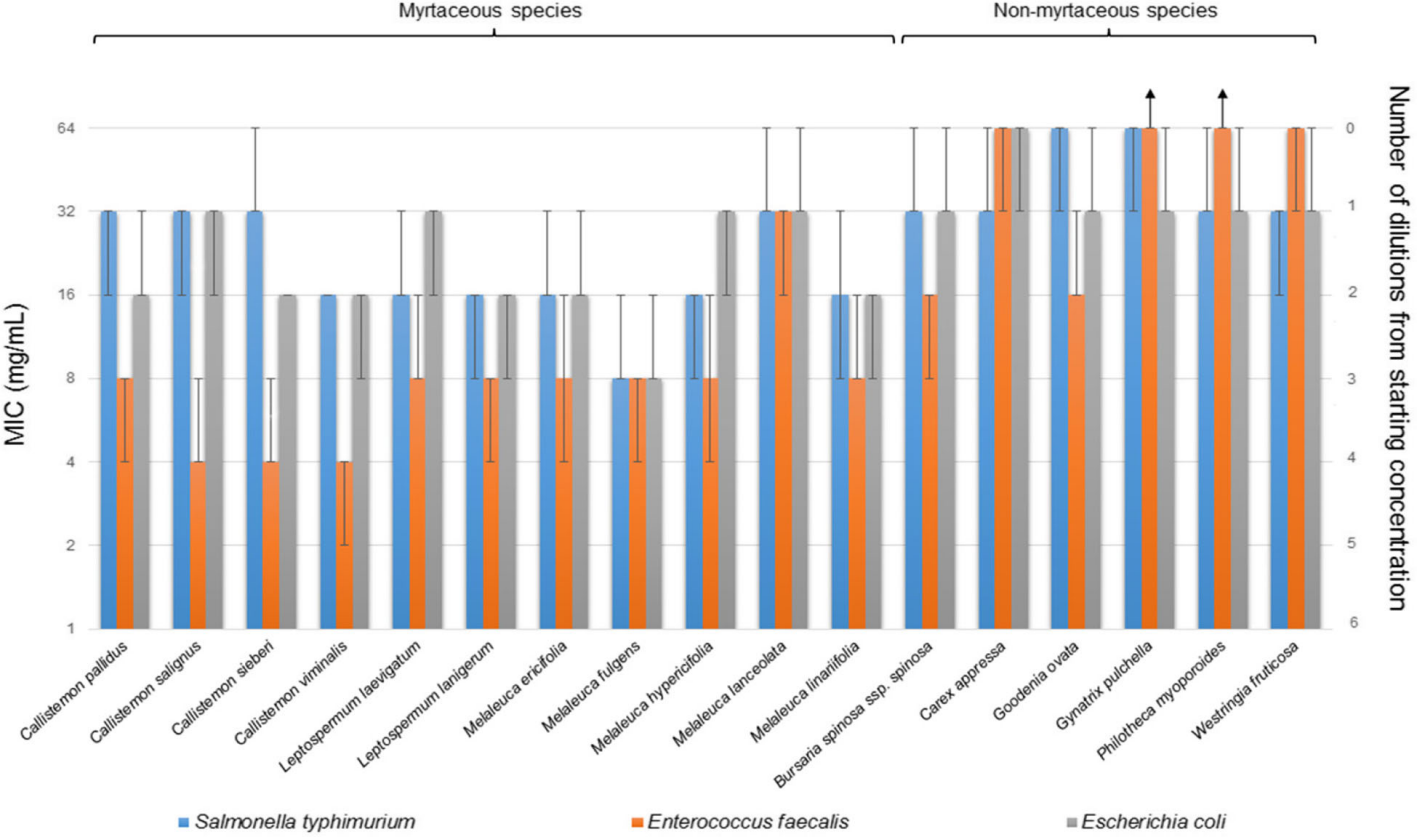
Median MICs of plant extracts against reference organisms. Blue, orange and grey bars represent the median MIC (mg/mL) for all replicates of tested plant species against *Salmonella* ser. Typhimurium*, E. faecalis* and *E. coli,* respectively. Error bars denote the range of MICs among replicates (*n* = 5 per plant species for each test organism, except for *G. ovata* where *n* = 6). Black arrows represent a range where ≥1 test replicate(s) of that species displayed MICs exceeding the upper testing limit (> 64 mg/mL), i.e. *G. pulchella* and *P. myoporoides* against *E. faecalis*

Of the list of 333 plant species compiled from Melbourne nurseries, 42 achieved overall species ranks of ≥3. In order to inform biofilter vegetation design guidelines for optimal microbial removal, species were categorised based on whether they demonstrated high (> 30) or low (< 1) antimicrobial scores. These were tentatively associated with stronger and weaker potentials for mediating antibiosis against faecal microorganisms within biofilters, respectively. All species demonstrating high antimicrobial scores (*n* = 11 species) belonged to the family Myrtaceae (tested genera: *Callistemon, Leptospermum* and *Melaleuca*), a family comprising species recognised for their significant production of antimicrobial oils [50, 65, 89] (listed in Fig. 1). These species were applied as “putatively antimicrobial” test plants. The remaining species (*n* = 6) were represented by a range of non-myrtaceous genera (*Philotheca myoporoides*, *Bursaria spinosa* subsp. *spinosa*, *Goodenia ovata*, *Westringia fruticosa* and *Carex appressa*). All of these lacked previous recognition of significant antimicrobial activity against human-derived microorganisms (antimicrobial scores ≤0.5; Additional file 1: Table S1). Accordingly, these were applied as “putatively non-antimicrobial” plants. Each selected species was sampled from diverse urban environments to account for the varying influences of intrinsic (e.g. genetic, plant life stage) and environmental (e.g. climate) factors on antimicrobial production. A total of 86 samples were collected (≥ 5 individuals per species). All selected species, their individual characteristics and scoring are described in Additional file 1: Table S1; GPS locations of all tested individuals are specified in Additional file 1: Tables S2 and Table S3.

### Antimicrobial susceptibility testing

Samples from selected species (*n* = 86 total) underwent processing for antimicrobial susceptibility testing (AST) against the selected reference organisms *E. coli, Salmonella* ser. Typhimurium and *E. faecalis*. The broth microdilution method was employed due to it being recognised as one of the most reproducible, economical, rapid and commonly employed methods for quantitative determination of plant antimicrobial activity [85, 90, 91]. Prior to testing, all extracts were confirmed free of culturable contaminant organisms, and to contain sufficient amounts of material allowing for resuspension at a starting concentration of 64 mg/mL. Consistent with previous observations, all 1% DMSO growth control wells (*n* = 2 per tray; 50 total) exhibited positive growth of all test organisms [45] indicating DMSO’s lack of inhibitory activity at 1% concentration. Furthermore, all 50 μg/mL gentamicin growth inhibition controls (n = 2 per tray; 50 total) demonstrated negative growth for all test organisms.

### Variability in antimicrobial activities between plant species

Resuspended methanolic extracts of all plants demonstrated MICs ranging from 2 to > 64 mg/mL (median: 16 mg/mL) against all three test organisms (Fig. 1. Median MICs and ranges for all test species are listed in Additional file 1: Table S2; individual sample MICs are tabulated in Additional file 1: Tables S3 and Table S4. Observed median MICs of tested plant extracts correlated with their Google Scholar literature-based antimicrobial scores (Table 1 & Additional file 1: Table S1; Spearman r = 0.622; Spearman’s rank test)**,** validating this method as a quick and easy means of screening for plant species’ antimicrobial potential.

Collectively, extracts of the “putatively antimicrobial” myrtaceous test plants demonstrated higher inhibitory activity against all tested organisms (median: 16 mg/mL, range: [2–64 mg/mL]) in comparison to the “putatively non-antimicrobial” non-myrtaceous counterparts (32 mg/mL, [8 - > 64 mg/mL]; *p* < 0.0001, uncorrected MW test). Significant variation was also observed between individual species (p < 0.0001, DKW test; all significant between-species comparisons are listed in Additional file 1: Table S5) (Fig. 1). All individual myrtaceous species, except for *M. lanceolata,* demonstrated greater inhibitory activity than the three lowest performing non-myrtaceous species (*p* < 0.05 for all DKW and PBMW comparisons), namely *G. pulchella*, *P. myoporoides* (both 64 mg/mL, [32 - > 64 mg/mL]) and *C. appressa* (64 mg/mL, [32–64 mg/mL]).

The three myrtaceous genera comprising “putatively antimicrobial” test plants, namely *Callistemon, Leptospermum* and *Melaleuca,* are well-recognised for their production of antimicrobial essential oils [92, 93]. Thus far, researchers have predominantly investigated essential oils and non-polar extracts in Australian plant AST studies [94]. The antimicrobial activity of polar extracts derived from these and other Australian plants remains considerably less explored, despite significant activity having been reported in existing studies [94, 95]. Extract solvent choice plays an important role in evaluating plant antimicrobial activity, owing to variation in solubility of different active compounds between solvents. Methanol is one of the most commonly employed polar solvents for AST of plant extracts [91], and is relatively straightforward, rapid, safe and economical to employ. Methanol has demonstrated high extraction efficiency of antimicrobial compounds from Australian plants [95], and allows enhanced diffusion of active compounds through aqueous microbial growth media relative to most other solvents. Given the benefits of investigating the activity of novel extracts of a species (e.g. broader characterisation of antimicrobial activity and secondary metabolome; potential discovery of new therapeutic agents lacking solubility in other solvents), further exploration of polar Australian plant extracts is warranted. Indeed, the present study provides the first report on the antimicrobial activity of polar extracts from multiple tested species.

*M. fulgens* consistently demonstrated the strongest activity against all tested bacteria (8 mg/mL, [4–16 mg/mL]), exhibiting significantly higher activity than all non-myrtaceous extracts (*p* < 0.02 for all DKW comparisons). In previous studies, *M. fulgens* essential oils have demonstrated insecticidal [96] and acaricidal activity [97], putatively attributed to their abundant 1,8-cineole and limonene content [96, 97]. The antimicrobial activity of *M. fulgens* had only been previously described for leaf essential oils [98], thus the present study represents the first report on the antimicrobial activity of *M. fulgens* polar extracts. The activity of *M. fulgens* extracts observed herein (4–16 mg/mL) is significantly lower than was observed for its essential oils (MIC range: 4–8 μg/mL) against a panel of similar test organisms including *E. coli*, *Salmonella enterica* (Gram-negative faecal stormwater organisms) and the skin pathogen *Staphylococcus aureus* (Gram-positive) [98]**.** This may be attributed to the relatively poor isolation of many terpenoids and other non-polar *M. fulgens* active compounds by methanolic extraction [94].

*C. viminalis* extracts demonstrated the most significant activity after *M. fulgens* (median MIC and range for combined organism MIC data: 16 mg/mL, [2–16 mg/mL]). This species has been relatively well-characterised for its antibacterial, antifungal and insecticidal properties, putatively attributed to multiple phenolics, alkaloids, triterpenoids, flavonoids and saponins [99]. Most notably, *C. viminalis* extracts demonstrated the strongest activity against any individual test organism, specifically *E. faecalis* (4 mg/mL, [2–4 mg/mL]). *Salmonella* ser. Typhimurium and *E. coli* demonstrated somewhat lower susceptibilities (16 mg/mL, [16 mg/mL] and 16 mg/mL, [8–16 mg/mL], respectively). Previous studies on foliar methanolic *C. viminalis* extracts yielded similar findings, with activity tending to be stronger against Gram-positive microorganisms (i.e. skin pathogen *Staphylococcus aureus* and foodborne disease bacterium *Bacillus cereus*; average MICs: 0.8 mg/mL), than Gram-negative organisms (i.e. *E. coli*, *Shigella sonnei* and *Pseudomonas aeruginosa*: 12.5 mg/mL; *Salmonella enteritidis*: 6.3 mg/mL) [100]. Notably, the activity of *C. viminalis* against the stormwater faecal microorganisms *E. coli* and *Salmonella sp*. observed by Delahaye et al. [100] (average MICs 12.5 and 6.3 mg/mL respectively) were very similar to the present findings (*E. coli* and *Salmonella* ser. Typhimurium MIC range: 8–16 mg/mL).

Also of interest was the high activity demonstrated by *L. lanigerum* (8 mg/mL, [4–16 mg/mL]). To our knowledge, no previous studies have characterised the antimicrobial activity of this species, notwithstanding biochemical profiles demonstrating significant levels of antimicrobial-associated secondary metabolites [101]. These include the monoterpenoids α-pinene (0.5–20% oil content), β-pinene (1–21%) [102], 1,8-cineole (3–7%) [103], linalool (0.4–3%) and α-terpineol (2–3%) [104], in addition to the sesquiterpenes β-caryophyllene (13 ± 57%) [105] and humulene (2 ± 22%) [106]. Given the observed similarity in activity of *L. lanigerum* in this study to the much better characterised *C. viminalis*, the antimicrobial activity of this species warrants further investigation.

The above three top-performing species exhibited significantly higher activities than all non-myrtaceous extracts (*P* < 0.02 for all non-myrtaceous vs. *M. fulgens*, *C. viminalis* and *L. lanigerum* comparisons; DKW test). Of key interest is that the current biofilter gold standard for pollutant removal, *Carex appressa,* demonstrated one of the lowest observed activities of tested plants against all reference microorganisms (64 mg/mL; [32–64 mg/mL]), up to eight-fold lower than the best performer *M. fulgens*. To our knowledge, this is the first report on the antimicrobial activity of *C. appressa*. There is little available information on its chemical constituents, although two compounds with potential antimicrobial activity, namely the aurone sulfuretin [107] and the flavonoid tricin [108], have been identified in small amounts in its tissues [109]. These or other unidentified compounds likely contributed to the observed sparing activity of *C. appressa* extracts.

*G. pulchella* and *P. myoporoides* exhibited the weakest activity of test candidates, notably against *E. faecalis* (> 64 mg/mL; [64 - > 64 mg/mL]), demonstrating only some modest activity against other test organisms (*P. myoporoides* and *G. pulchella* against *E. coli*: 32 mg/mL, [32–64 mg/mL]; *P. myoporoides* against *Salmonella* ser. Typhimurium: 32 mg/mL, [32–64 mg/mL]). *G. pulchella* has not been previously characterised for its antimicrobial activity or metabolites. The sparing activity observed for *P. myoporoides* extracts may be attributed to its production of small amounts of metabolites with established antimicrobial (e.g. the terpenoid α-pinene [110]) or putatively antimicrobial (e.g. sesquiterpenyl coumarins and geranyl benzaldehyde derivatives) activity [110, 111]. The low overall activity of *P. myoporoides* is consistent with a previous AST study, where a 4 mg/mL methanolic leaf extract was observed to lack significant inhibition of similar organisms (*E. coli* and *Salmonella* ser. Typhimurium) among others [94].

Unexpectedly, *M. lanceolata* demonstrated significantly lower activity compared to other tested myrtaceous plants (32 mg/mL, [16–64 mg/mL]; *p* < 0.0001, uncorrected MW test for *M. lanceolata* vs. combined myrtaceous plant data). Previous investigations on the activity of this species remain limited, although its essential oils have reported insecticidal activity [96] and moreover contain various antimicrobial-associated compounds (1,8-cineole, globulol, sesquiterpenes and α-pinene) [112]. Aside from organism-specific differences in susceptibility (insecticidal vs. bactericidal), extraction method may have accounted for the low inhibitory activity observed in the present study. Polar extracts may underrepresent the activity of plants containing significant proportions of non-polar antimicrobial compounds. Indeed, a similar lack of antimicrobial activity was observed for polar (aqueous) *M. lanceolata* extracts in a previous study [113]. Furthermore, single extracts may not reflect complex synergistic/antagonistic effects against microorganisms by plants *in natura*. This is a reminder that the present study solely provides a rapid screening method for estimating plant antimicrobial activity; multiple AST methods are recommended to provide more holistic antimicrobial characterisation.

### Variability in antimicrobial activities between test organisms

Myrtaceous plant extracts consistently exhibited comparatively strong activity against the Gram-positive bacterium *E. faecalis* (8 mg/mL, [2–32 mg/mL]) than against Gram-negative organisms *Salmonella* ser. Typhimurium and *E. coli* (*p* < 0.0001 for individual DKW and combined MW MIC data comparisons). The latter two organisms, both members of the *Enterobacteriaceae*, demonstrated high similarity in their MIC distributions (median and range of both: 16 mg/mL, [8–64 mg/mL]; *p* > 0.9999; DKW test between *E. coli* and *Salmonella* ser. Typhimurium total data), likely attributed to their high genetic relatedness [114]. *E. faecalis* did not demonstrate significantly greater susceptibility to non-myrtaceous *W. fruticosa, P. myoporoides, G. pulchella* and *C. appressa* extracts, suggesting a comparative lack of antimicrobial compounds produced by these plants with *Enterococcus-* or Gram-positive-specific activity.

The relative susceptibility of Gram-positive to Gram-negative bacteria against plant extracts has been observed previously. The comparative resistance of Gram-negative organisms has been explained by their possession of an outer membrane, which acts as an effective barrier to amphipathic antimicrobial compounds [115–117]. Overexpressed or multiple efflux pumps in Gram-negative bacteria have moreover been associated with enhancing the transport of antimicrobial substances out of the cell [118, 119]. More specifically, *Enterobacteriaceae* are often reported to demonstrate greater inhibition to myrtaceous plant extracts than enterococci [120–123]. This has been putatively ascribed to abundant polyphenol production in myrtaceous plants, which can facilitate cytoplasmatic membrane damage and inhibit the synthesis of cell walls, cell membranes and nucleic acids [120]. Nevertheless, the specific identities of Gram-positive active compounds isolated from polar Australian myrtaceous plant extracts remain vastly uncharacterised [124].

### Intra-species variability in antimicrobial activity

Aside from inter-species variation, differences in extract activity were occasionally observed between unique biological individuals derived from the same species. Equivalent or very similar activities were observed for individual replicates of the same species (MICs ±1 dilution of each other for all test organisms), with the exception of *M. ericifolia*, *M. hypericifolia* (both 4–16 mg/mL for *E. faecalis*) and *M. linariifolia* (8–32 mg/mL for *Salmonella* ser. Typhimurium), demonstrating up to four-fold differences in activity between replicates (MICs ±2 dilutions of each other; all MICs listed in Additional file 1: Table S3). Observed disparities in activity between biological replicates remained consistent despite confirmatory testing on new, independently prepared extracts from the same plants.

Individuals of the same species often demonstrate equivalent or very similar activities even when cultivated in distinct environments, as was observed for most candidates in the present study [125–127]. However, inconsistencies in activity can arise due to differing levels of exposure to various biotic and abiotic stresses. Indeed, applying various stressing conditions has established efficacy for enhancing secondary metabolite production, including antimicrobials, in plants [54]. Upon further investigation, the *M. hypericifolia* individual demonstrating the lowest activity against *E. faecalis* (MIC = 16 mg/mL) was situated in a more shaded, higher altitude site with higher rainfall relative to other sampling locations (Karwarra Garden, Mt. Dandenong, 420–440 m AMSL, mean annual rainfall: 1262.3 mm). Conversely, the *M. hypericifolia* individual with the highest *E. faecalis* inhibitory activity (MIC = 4 mg/mL) was situated in a much drier, lower altitude location (Maranoa Gardens, Balwyn, ~ 100 m AMSL, mean annual rainfall: 686.5 mm). A corresponding trend was observed among the most and least active *M. linariifolia* replicates against *Salmonella* ser. Typhimurium (MIC = 8 mg/mL vs. 32 mg/mL), with the former being planted in a drier, more exposed location than the latter. Similar observations have been recorded previously, where individuals grown in drier [56, 57] and hotter locations have demonstrated higher antimicrobial production than those from lower altitude sites with cooler, wetter climates [58]. Changes in antimicrobial production under water stress may be particularly relevant in the context of biofilters, where plants commonly withstand extended drying periods. Moisture, temperature or other unmonitored parameters such as UV exposure, soil nutrient levels, intrinsic plant factors (e.g. age, genetic factors/chemotype) and predation/infection by pathogens may have accounted for observed activity disparities between replicates. The differential production of antimicrobial compounds between unique individuals under varying operational conditions may have significant implications for vegetation-mediated pathogen removal within biofilters.

### Vegetation selection for antimicrobial biofilters

The results of this study suggest that many myrtaceous species, particularly *M. fulgens, C. viminalis* and *L. lanigerum*, may enhance biofilter-mediated pathogen removal relative to *C. appressa* and other non-myrtaceous plants. Indeed, certain *Melaleuca* and *Leptospermum* species have previously demonstrated high faecal microorganism removal in stormwater biofilters [22] and *E. coli*-contaminated soil [65]. While these top performing species have been recommended by various biofilter vegetation selection guidelines based on practicality and survivability [69], they have not been specifically identified for enhancing pollutant removal. We recommend further investigation of the *in natura* antimicrobial activity of these species against multiple stormwater pathogens within biofilters. In contrast, other species in this paper demonstrated poor activity relative to other candidates, and may be cautiously advised against for antimicrobial vegetation selection.

In line with previous research, the results of this study reveal varying susceptibilities of different microorganisms to plant extracts. This highlights the challenge in selecting universally effective vegetation for the removal of all stormwater pathogens, particularly Gram-negative bacteria. Many important stormwater faecal pathogens are Gram-negative, including *Salmonella* sp., *Campylobacter* sp. and pathogenic *E. coli* O157:H7. Further research is required to determine the efficacy of plant-mediated antimicrobial treatment of viral, protozoan and other bacterial stormwater pathogens in vitro and within biofilters. Indeed, the inherently differing susceptibilities of stormwater microorganisms to different plant antimicrobials raises the case for incorporating mixed plant communities into biofilters for multilateral treatment.

Significant four-fold differences between species individuals were occasionally observed. Intrinsic and environmental factors influencing plant secondary metabolism inherently differ within (temporally) and between biofilters depending on specific system design (e.g. incorporation of a SZ, filter media type) and operational conditions (e.g. surrounding land use, seasonal, climatic and hydrologic factors). The differential influence of these variables on the quality and quantity of plant antimicrobials deposited into biofilters may affect the extent of vegetation-mediated pathogen removal. Addressing the current knowledge gaps surrounding this phenomenon will inform vegetation guidelines for optimal performance. More broadly, while not being common practice in plant AST studies, our findings highlight the importance of testing multiple biological replicates from diverse environments to adequately reflect natural variability in antimicrobial activity of a given species. Incorporating routine multi-replicate testing is thus strongly recommended for strengthening method design in prospective studies within plant pharmacology.

## Conclusions

The methanolic leaf extracts of 17 plant species, selected for their suitability in south-eastern Australian stormwater biofilters, were investigated for their antimicrobial activities against common stormwater bacteria *E. coli, Salmonella* ser. Typhimurium and *E, faecalis*. The employed selection and testing method was validated as a safe, simple, rapid and inexpensive preliminary screening approach to select biofilter-suitable plant species with antimicrobial activity (based on a Melbourne case study).

Our results suggest that myrtaceous plants, particularly *M. fulgens, C. viminalis* and *L. lanigerum*, may enhance pathogen inactivation within biofilters relative to poorer performing non-myrtaceous plants. Notably, these species are predicted to demonstrate enhanced pathogen treatment relative to the current biofilter vegetation gold standard, *C. appressa*. Further investigation of these high-performing species for their pathogen removal in biofilter contexts is recommended. Notably, the activity of plant species often varied against different microorganisms. This suggests that integrating multiple high-performing plants into biofilters may achieve optimal pathogen killing efficacy.

Occasional significant differences in activity were observed between different species replicates, suggesting that biofilter operational conditions (e.g. extended drying periods, high temperatures, high UV exposure) may influence plant antimicrobial production and thus overall treatment. Testing multiple genetically and environmentally diverse individuals appears to be essential for accurate antimicrobial characterisation of a species. This practice is uncommon in plant AST studies and thus forms an important recommendation of this research.

On a broader note, further research into the activity of these plant species against other clinically significant microorganisms, particularly under-researched candidates like *L. lanigerum*, may elucidate candidates for future development of novel antimicrobial agents.

## Additional file

Additional file 1: Complete experimental data for selected test plant species. (DOCX 106 kb)

## Abbreviations

AST: antimicrobial susceptibility testing
CFU: colony forming unit(s)
DKW: Dunn’s corrected Kruskal-Wallis
DMSO: dimethyl sulfoxide
*E. coli*: *Escherichia coli*
*E. faecalis*: *Enterococcus faecalis*
FIO: Faecal indicator organism
INT: 2-p-iodophenyl-3-p-nitrophenyl-5-phenyl tetrazolium chloride
MHB: Mueller-Hinton broth
MW: Mann-Whitney
OD: optical density
PBMW: *post hoc* Bonferroni-corrected Mann-Whitney
rpm: rotations per minute
*Salmonella* ser. Typhimurium: *Salmonella enterica* subsp. *enterica* ser. Typhimurium
SZ: submerged zone
WSUD: Water sensitive urban design
