# Supplementary material for: Rise of the killer plants: investigating the antimicrobial activity of Australian plants to enhance biofilter-mediated pathogen removal
Source: J Biol Eng. 2019 Jun 6;13:52. doi: 10.1186/s13036-019-0175-2 (PMC6555726; doi:10.1186/s13036-019-0175-2)
Supplement: Supplementary file 1 — Complete experimental data for selected test plant species. (DOCX 106 kb) [file 13036_2019_175_MOESM1_ESM.docx]

## Additional File 1

**Table S1.** Complete and detailed characteristics of selected plant species demonstrating overall species ranks of ≥ 3 where n ≥ 5 individuals that were successfully sampled in urban areas of southern Victoria.

| Species | Available from Melbourne nurseries | Anti-microbial activity rank | Secondary metabolite rank | Overall anti-microbial score | Adaptation to key biofilter conditions | Adaptation score (1-3) | Root structure | Root score | Weed potential (low/medium/  high risk) | Height of mature plant (m) | Canopy width of mature plant (m) | Woody plant | Indigenous to Melbourne | Past successful application in biofilters | Growth rate | Nitrogen-fixation | Lifespan | Overall species rank |
| --- | --- | --- | --- | --- | --- | --- | --- | --- | --- | --- | --- | --- | --- | --- | --- | --- | --- | --- |
| Putatively antimicrobial test plants | | | | | | | | | | | | | | | | | | |
| *Melaleuca ericifolia* | Yes | 47 | 240 | 167 | Hardy in most soils and situations. Sandy soil-tolerant; very waterlogging-tolerant; tolerant of hot, dry conditions in Melbourne’s summer | 3 | Extensive root system with fine roots [1, 2] | 3 | Low | 2-9 | 2-6 | Yes | Indigenous to Melbourne | High nitrogen and E. coli removal observed in biofilters planted with Melaleuca sp. [3-5] | Fast-growing | Non-N2-fixing | Long-lived | 5 |
| *Melaleuca fulgens* | Yes | 47 | 240 | 167 | Hardy in most soils and situations. Sandy soil-tolerant; tolerant of temporary waterlogging; tolerant of hot, dry conditions in Melbourne’s summer | 3 | Extensive, fine roots | 3 | Low | 1-3 | 1.5 – 3 | Yes | Not indigenous but native to Australia; suitable for growing in Melbourne | High nitrogen and E. coli removal observed in biofilters planted with Melaleuca sp. [3-5] | Fast-growing | Non-N2-fixing | Long-lived | 4.8 |
| *Melaleuca hypericifolia* | Yes | 47 | 240 | 167 | Hardy in most soils and situations. Sandy soil-tolerant; tolerant of temporary waterlogging; tolerant of hot, dry conditions in Melbourne’s summer | 3 | Extensive, fine roots | 3 | Low | 2-5 | 2.5 | Yes | Not indigenous but native to Australia; suitable for growing in Melbourne | High nitrogen and E. coli removal observed in biofilters planted with Melaleuca sp. [3-5] | Fast-growing | Non-N2-fixing | Long-lived | 4.8 |
| *Melaleuca lanceolata* | Yes | 47 | 240 | 167 | Hardy in most soils and situations. Sandy soil-tolerant; tolerant of temporary waterlogging; tolerant of hot, dry conditions in Melbourne’s summer | 3 | Extensive, fine roots | 3 | Low-Medium; May become invasive in some areas | 4-8 | 3-6 | Yes | Indigenous to Melbourne | High nitrogen and E. coli removal observed in biofilters planted with Melaleuca sp. [3-5] | Fast-growing | Non-N2-fixing | Long-lived (over 15 years) | 5 |
| *Melaleuca linariifolia* | Yes | 47 | 240 | 167 | Hardy in most soils and situations. Sandy soil-tolerant; tolerant of temporary waterlogging; tolerant of hot, dry conditions in Melbourne’s summer | 3 | Extensive, fine roots | 3 | Low | 1.5 – 10 | 1.00 – 1.50 | Yes | Not indigenous but native to Australia; suitable for growing in Melbourne | High nitrogen and E. coli removal observed in biofilters planted with Melaleuca sp. [3-5] | Fast-growing | Non-N2-fixing | Long-lived | 4.8 |
| *Callistemon little john* | Yes | 41 | 79 | 80.5 | Hardy in most soils and situations. Sandy soil-tolerant; tolerant of temporary waterlogging; tolerant of hot, dry conditions in Melbourne’s summer | 3 | Extensive, fine roots | 3 | Low | 1.5 | 1 – 1.5 | Yes | Not indigenous but native to Australia; suitable for growing in Melbourne | N/A | Fast-growing | Non-N2-fixing | Long-lived | 3.8 |
| *Callistemon citrinus* | Yes | 41 | 79 | 80.5 | Hardy in most soils and situations. Sandy soil-tolerant; tolerant of temporary waterlogging; tolerant of hot, dry conditions in Melbourne’s summer | 3 | Extensive, fine roots | 3 | Low | 2 – 2 | 2.00 – 2.00 | Yes | Not indigenous but native to Australia; suitable for growing in Melbourne | N/A | Fast-growing | Non-N2-fixing | Long-lived | 3.8 |

| Species | Available from Melbourne nurseries | Anti-microbial activity rank | Secondary metabolite rank | Overall anti-microbial score | Adaptation to key biofilter conditions | Adaptation score (1-3) | Root structure | Root score | Weed potential (low/medium/  high risk) | Height of mature plant (m) | Canopy width of mature plant (m) | Woody plant | Indigenous to Melbourne | Past application and successful use in biofilters | Growth rate | Nitrogen-fixation | Lifespan | Overall species rank |
| --- | --- | --- | --- | --- | --- | --- | --- | --- | --- | --- | --- | --- | --- | --- | --- | --- | --- | --- |
| *Callistemon pallidus* | Yes | 41 | 79 | 80.5 | Hardy in most soils and situations. Sandy soil-tolerant; tolerant of temporary waterlogging; tolerant of hot, dry conditions in Melbourne’s summer | 3 | Extensive, fine roots | 3 | Low | 1.5 – 2 | 1.00 – 1.50 | Yes | Not indigenous but native to Australia; suitable for growing in Melbourne | N/A | Fast-growing | Non-N2-fixing | Long-lived | 3.8 |
| *Callistemon salignus* | Yes | 41 | 79 | 80.5 | Hardy in most soils and situations. Sandy soil-tolerant; tolerant of temporary waterlogging; tolerant of hot, dry conditions in Melbourne’s summer | 3 | Extensive, fine roots | 3 | Low | 2 – 3 | 2.00 – 3.00 | Yes | Not indigenous but native to Australia; suitable for growing in Melbourne | N/A | Fast-growing | Non-N2-fixing | Long-lived | 3.8 |
| *Callistemon sieberi* | Yes | 41 | 79 | 80.5 | Hardy in most soils and situations. Sandy soil-tolerant; tolerant of temporary waterlogging; tolerant of hot, dry conditions in Melbourne’s summer | 3 | Extensive, fine roots | 3 | Low | 3-10 | 2-6 | Yes | Indigenous to Melbourne | N/A | Fast-growing | Non-N2-fixing | Long-lived | 4 |
| *Leptospermum continentale* | Yes | 31 | 46 | 54 | Hardy in most soils and situations. Sandy soil-tolerant; tolerant of temporary waterlogging; tolerant of hot, dry conditions in Melbourne’s summer | 3 | Deep, extensive root system with fine roots [5] | 3 | Low | 1-4 | 1-2 | Yes | Indigenous to Melbourne | High E. coli removal [5, 6] and high nitrogen removal in wet conditions [7] | Fast-growing | Non-N2-fixing | Long-lived | 4.3 |
| *Leptospermum laevigatum* | Yes | 31 | 46 | 54 | Hardy in sandy soils; poor tolerance of prolonged waterlogging, however temporary waterlogging in biofilters is likely not problematic; tolerant of hot, dry conditions in Melbourne’s summer; not waterlogging tolerant but should be tolerant of periodic inundation | 2.5 | Extensive, fine roots | 3 | Low-Medium; May become invasive in some areas | 2-8 | 2-4 | Yes | Indigenous to Melbourne | Likely to be effective; high E. coli removal [5, 6] and high nitrogen removal in wet conditions [7] by other *Leptospermum sp.* | Fast-growing | Non-N2-fixing | Long-lived | 4.2 |
| *Leptospermum lanigerum* | Yes | 31 | 46 | 54 | Hardy in many situations. Sandy soil-tolerant; tolerant of temporary waterlogging; tolerant of hot, dry conditions in Melbourne’s summer | 3 | Deep, extensive root system with fine roots | 3 | Low | 2-6 | 1-3 | Yes | Indigenous to Melbourne | Likely to be effective; high E. coli removal [5, 6] and high nitrogen removal in wet conditions [7] by other *Leptospermum sp.* | Fast-growing | Non-N2-fixing | Long-lived | 4.3 |

| Species | Available from Melbourne nurseries | Anti-microbial activity rank | Secondary metabolite rank | Overall anti-microbial score | Adaptation to key biofilter conditions | Adaptation score (1-3) | Root structure | Root score | Weed potential (low/medium/  high risk) | Height of mature plant (m) | Canopy width of mature plant (m) | Woody plant | Indigenous to Melbourne | Past application and successful use in biofilters | Growth rate | Nitrogen-fixation | Lifespan | Overall species rank |
| --- | --- | --- | --- | --- | --- | --- | --- | --- | --- | --- | --- | --- | --- | --- | --- | --- | --- | --- |
| Putatively non-antimicrobial test plants | | | | | | | | | | | | | | | | | | |
| *Philotheca myoporoides* | Yes | 0 | 1 | 0.5 | Hardy shrub. Sandy soil-tolerant; tolerant of temporary waterlogging; tolerant of hot, dry conditions in Melbourne’s summer | 2.5 | Fairly shallow-rooted with some fine roots | 1.5 | Low | 1 – 2 | 1.00 – 2.00 | Yes | Not indigenous but native to Australia; suitable for growing in Melbourne | N/A | Fast-growing | Non-N2-fixing | Long-lived | 3.8 |
| *Bursaria spinosa ssp. Spinosa* | Yes | 0 | 0 | 0 | Fairly hardy shrub. Sandy soil-tolerant; tolerant of temporary waterlogging; tolerant of hot, dry conditions in Melbourne’s summer | 2.5 | Strong, fibrous root system | 2.5 | Low | 2-6 | 2-3 | Yes | Indigenous to Melbourne | N/A | Medium growth rate | Non-N2-fixing | Long-lived | 4.2 |
| *Goodenia ovata* | Yes | 0 | 0 | 0 | Hardy shrub. Sandy soil-tolerant; tolerant of temporary waterlogging; tolerant of hot, dry conditions in Melbourne’s summer | 3 | Roots are long and fine [1, 2] | 2.5 | Low | 1-2.5 | 1-3 | Semi-woody | Indigenous to Melbourne | High nitrogen and phosphorous removal [1, 2] | Fast-growing | Non-N2-fixing | Short-lived (~8 years) | 4.7 |
| *Gynatrix pulchella* | Yes | 0 | 0 | 0 | Hardy shrub. Sandy soil-tolerant; tolerant of temporary waterlogging; tolerant of hot, extended dry conditions in Melbourne’s summer but not prolonged drought | 2.5 | Long thick tap root lacking extensive fine root system | 1 | Low | 2-4 | 1.5-3 | Yes | Indigenous to Melbourne | N/A | Fast-growing | Non-N2-fixing | Short-lived | 3.7 |
| *Westringia fruticosa* | Yes | 0 | 0 | 0 | Hardy in most soils and situations. Sandy soil-tolerant; tolerant of temporary but not extended waterlogging; tolerant of hot, dry conditions in Melbourne’s summer | 3 | Thick tap root lacking extensive fine root system | 1 | Low | 1.5 – 2 | 1.5 – 2 | Yes | Not indigenous but native to Australia; suitable for growing in Melbourne | N/A | Fast-growing | Non-N2-fixing | Fairly long-lived | 3.8 |
| *Carex appressa* | Yes | 0 | 1 | 0.5 | Hardy in sandy soils; tolerant of temporary waterlogging; tolerant of hot, dry conditions in Melbourne’s summer | 3 | Extensive fine roots [1, 2] | 3 | Low | 0.5 – 1.2 | 0.5 – 1 | No | Indigenous to Melbourne | High nitrogen and phosphorous removal [1, 2] | Fast-growing | Non-N2-fixing | Fairly long-lived | 4.8 |

Test plants were selected from collated nursery stock lists where they met multiple criteria to yield overall species ranks of ≥ 3. Species that were successfully identified and sampled ≥ 5 times in the field are listed above in Table S.1. All samples were collected during Melbourne’s spring period, 2016 (29/09/16-17/11/16). All species descriptions refer to those of mature plants. Prior to ranking, species were excluded from this original list if they posed a high “weed potential”, or risk of becoming invasive. Further, species typically growing > 10 m in height and < 1 m in canopy diameter (sedges excepted) were excluded prior to ranking.

Overall species ranks were assigned to all remaining listed plants based on their overall antimicrobial scores, adaptation to key biofilter conditions, root structure, woody/herbaceous physiology, indigeneity to Melbourne, past successful application/performance in biofilters, growth rate, nitrogen-fixing capability and lifespan. Each criterion was informed by literature [1, 2, 4, 5, 8-22], in the absence of which information was sourced from experienced horticulturalists including Evan Cluclas (personal communication, 2016; owner of Karunga native nursery) and others from major native plant nurseries around Melbourne (VINC, Knox, Braeside, Whitehorse, Box Hill, Ringwood, SKINK, Bushland Flora) from which species lists were derived. The total sums of all individual scores assigned to each species for each criterion were divided by 3 to generate final species ranks. Species demonstrating overall species ranks < 2 were excluded from selection. These criteria and their contributions to overall species ranks are delineated as follows.

**Antimicrobial score:** Overall antimicrobial scores were assigned to provide an indication of known antimicrobial activity using the Google Scholar academic literature search engine (<http://scholar.google.com.au>, USA). Species were assigned an antimicrobial score based on the number of positive Google Scholar search results associating plant genus with antimicrobial-associated terms. Antimicrobial scores were combined of two parts. 1) For each listed species an antimicrobial activity rank was assigned based on the recorded number of publications retrieved on Google Scholar with titles including both the name of the selected plant’s genus with one or more antimicrobial-associated term(s) (search syntax: allintitle: *genus name* antimicrobial OR antibiotic -streptomyces -actinomycete -bacteria -fungi). 2) Publications were then screened for titles which included the name of the selected plant’s genus in addition to secondary metabolite groups associated with antimicrobial activity [8-12] (search syntax: allintitle: *genus name* "essential oil" OR "essential oils" OR phenol OR flavonoid OR defensins OR “proteinase inhibitor” OR “cyanogenic glycosides” OR anthocyanin OR quinone OR polyphenol OR terpenoid OR alkaloid). This number was divided by two to reflect its lesser importance relative to part 1 (i.e. publications directly related to antimicrobial activity). This value represented the secondary metabolite rank of each candidate. Parts 1 and 2 were summed to yield the overall antimicrobial score for each plant. Plants demonstrating very low or high antimicrobial scores (< 1 or > 30) were considered for further selection as “putatively antimicrobial” and “putatively non-antimicrobial” test plants, respectively. Antimicrobial scores of >150 contributed 5 points towards the species’ “overall species rank”; scores of 100-150 contributed 4 points; scores of 50-99 contributed 3 points; scores of 30-49 contributed 2 points; and antimicrobial scores <1 contributed 5 points.

**Adaptation to key biofilter conditions**: species were scored (1 - 3) based on their ability to maintain healthy growth under south-eastern Australian biofilter conditions (i.e. survival in sandy soil with temporary inundation and extended hot, dry periods). Plants demonstrating all the key traits associated with good survival and growth in biofilter conditions (i.e. hardy in sandy soils; tolerant of temporary waterlogging; tolerant of hot, dry conditions in Melbourne’s summer) were assigned an “adaptation score” of 3. Plants that demonstrated poor drought tolerance, poor growth in sandy soil, poor tolerance of waterlogging and poor tolerance of hot, dry conditions were assigned a score of 0. Plants demonstrating some “good” traits but some “poor” traits were assigned a score of 1 or 2 depending on the proportional ratio of good to poor traits (all plant traits listed in “Adaptation to key biofilter conditions” column). Plants with adaptation scores of ≥ 2 were considered for further selection, while others with scores < 2 were eliminated.

**Root structure:** Plants with fine, extensive roots were preferentially selected based on their tendency to demonstrate superior pollutant removal in biofilters [1, 5, 15]. Species were assigned a root score of 1, 2 or 3 based on root structure characteristics, with 3 representing “very good” (deep, dense, extensive, fine roots), 2 representing “average” and 1 representing “poor” roots (shallow, thick, minimal root systems). Root physiologies of all species are outlined in the “Root structure” column. Candidates with scores ≥ 2 were considered for selection, while others with scores < 2 were eliminated.

**Woody/herbaceous physiology**: Woody species were preferentially selected over herbaceous plants owing to their propensity to live longer, root more extensively, grow taller and produce more biomass and leaf litter for improved treatment capacity [13, 14]. Woody plants were assigned 0.5 points towards their overall species rank, while non-woody/herbaceous plants were allocated 0 points.

**Indigeneity to Melbourne**: Species indigenous to Melbourne were preferentially selected over other Australian natives and were assigned 0.5 points towards their overall species rank, while non-indigenous plants were allocated 0 points.

**Past successful application/performance in biofilters:** Species with high past performance in biofilters were preferentially selected and were assigned 1 point towards their overall species rank, while others were allocated 0 points.

**Growth rate:** Species with high growth rates were preferentially selected due to their association with improved nutrient removal [1, 15]. Plants demonstrating fast/medium/slow growth rates were assigned scores of 3, 2 and 1 respectively; these were divided by 3 before being added to the species’ overall rank to reflect the lower relative weighting of this parameter.

**Nitrogen-fixing capability:** Species lacking nitrogen-fixing root systems were preferentially selected to avoid compromised nitrogen removal [2, 15]. Species were designated as nitrogen-fixing or non-nitrogen-fixing and were assigned scores of 0 and 0.5 respectively towards their overall species ranks.

**Lifespan**: Plants with lifespans > 20 years were preferentially selected over shorter-lived species requiring frequent replacement. Plant lifespan was designated as long-lived (>20 years) or short-lived (< 20 years) and were assigned scores of 0.5 and 0 respectively towards their overall species ranks.

**Table S2:** Statistical mean and range values for minimum inhibitory concentrations for all plant species against tested microorganisms

| Plant species | Median *Salmonella* ser. Typhimurium MIC (mg/mL) | *Salmonella* ser. Typhimurium MIC range [min, max] (mg/mL) | Median *E. faecalis* MIC (mg/mL) | *E. faecalis* MIC range [min, max] (mg/mL) | Median *E. coli* MIC (mg/mL) | *E. coli* MIC range [min, max] (mg/mL) | Median MIC for all test organisms (mg/mL) | MIC range [min, max] (mg/mL) |
| --- | --- | --- | --- | --- | --- | --- | --- | --- |
| *Callistemon pallidus* | 32 | [16, 32] | 8 | [4, 8] | 16 | [16, 32] | 16 | [4, 32] |
| *Callistemon salignus* | 32 | [16, 32] | 4 | [4, 8] | 32 | [16, 32] | 16 | [4, 32] |
| *Callistemon sieberi* | 32 | [32, 64] | 4 | [4, 8] | 16 | [16, 16] | 16 | [4, 64] |
| *Callistemon viminalis (Little John cultivar)* | 16 | [16, 16] | 4 | [2, 4] | 16 | [8, 16] | 16 | [2, 16] |
| *Leptospermum laevigatum* | 16 | [16, 32] | 8 | [8, 16] | 32 | [16, 32] | 16 | [8, 32] |
| *Leptospermum lanigerum* | 16 | [8, 16] | 8 | [4, 8] | 16 | [8, 16] | 8 | [4, 16] |
| *Melaleuca ericifolia* | 16 | [16, 32] | 8 | [4, 16] | 16 | [16, 32] | 16 | [4, 32] |
| *Melaleuca fulgens* | 8 | [8, 16] | 8 | [4, 8] | 8 | [8, 16] | 8 | [4, 16] |
| *Melaleuca hypericifolia* | 16 | [8, 16] | 8 | [4, 16] | 32 | [16, 32] | 16 | [4, 32] |
| *Melaleuca lanceolata* | 32 | [32, 64] | 32 | [16, 32] | 32 | [32, 64] | 32 | [16, 64] |
| *Melaleuca linariifolia* | 16 | [8, 32] | 8 | [8, 16] | 16 | [8, 16] | 16 | [8, 32] |
| *Bursaria spinosa ssp. spinosa* | 32 | [32, 64] | 16 | [8, 16] | 32 | [32, 64] | 32 | [8, 64] |
| *Carex appressa* | 32 | [32, 64] | 64 | [32, 64] | 64 | [32, 64] | 64 | [32, 64] |
| *Goodenia ovata* | 64 | [32, 64] | 16 | [16, 32] | 32 | [32, 64] | 32 | [16, 64] |
| *Gynatrix pulchella* | 64 | [32, 64] | >64 | [64, >64] | 32 | [32, 64] | 64 | [32, >64] |
| *Philotheca myoporoides* | 32 | [32, 64] | >64 | [64, >64] | 32 | [32, 64] | 64 | [32, >64] |
| *Westringia fruticosa* | 32 | [16, 32] | 64 | [32, 64] | 32 | [32, 64] | 32 | [16, 64] |

MIC defines the minimum inhibitory concentration (mg/mL) of methanolic leaf extracts derived from each test plant against selected test microorganisms

**Table S3:** Minimum inhibitory concentration and location data for all replicates of “putatively antimicrobial” test plants

| Family | Scientific name | Common name | *Salmonella* ser. Typhimurium MIC (mg/mL) | *E. faecalis* MIC (mg/mL) | *E. coli* MIC (mg/mL) | Collection location (GPS coordinates) |
| --- | --- | --- | --- | --- | --- | --- |
| Myrtaceae | *Callistemon pallidus* | Lemon bottlebrush | 32 | 4 | 16 | -37.811, 145.0897 |
| Myrtaceae | *Callistemon pallidus* | Lemon bottlebrush | 16 | 8 | 32 | -37.8096, 145.0909 |
| Myrtaceae | *Callistemon pallidus* | Lemon bottlebrush | 32 | 8 | 16 | -37.8159, 145.3807 |
| Myrtaceae | *Callistemon pallidus* | Lemon bottlebrush | 32 | 8 | 16 | -38.1292, 145.2681 |
| Myrtaceae | *Callistemon pallidus* | Lemon bottlebrush | 16 | 4 | 16 | -37.8294, 144.9832 |
| Myrtaceae | *Callistemon salignus* | Willow bottlebrush | 32 | 8 | 32 | -37.9086, 145.139 |
| Myrtaceae | *Callistemon salignus* | Willow bottlebrush | 32 | 4 | 32 | -37.811, 145.09 |
| Myrtaceae | *Callistemon salignus* | Willow bottlebrush | 32 | 4 | 32 | -37.8296, 145.0209 |
| Myrtaceae | *Callistemon salignus* | Willow bottlebrush | 16 | 4 | 16 | -38.1294, 145.268 |
| Myrtaceae | *Callistemon salignus* | Willow bottlebrush | 16 | 8 | 16 | -37.8318, 144.9778 |
| Myrtaceae | *Callistemon sieberi* | River bottlebrush | 32 | 8 | 16 | -37.811, 145.0899 |
| Myrtaceae | *Callistemon sieberi* | River bottlebrush | 32 | 4 | 16 | -37.7899, 145.037 |
| Myrtaceae | *Callistemon sieberi* | River bottlebrush | 32 | 4 | 16 | -37.6919, 144.5901 |
| Myrtaceae | *Callistemon sieberi* | River bottlebrush | 64 | 4 | 16 | -37.7159, 145.0493 |
| Myrtaceae | *Callistemon sieberi* | River bottlebrush | 32 | 8 | 16 | -37.8282, 144.9801 |
| Myrtaceae | *Callistemon viminalis* | Creek bottlebrush "Little John" cultivar | 16 | 4 | 16 | -37.8103, 145.091 |
| Myrtaceae | *Callistemon viminalis* | Creek bottlebrush "Little John" cultivar | 16 | 4 | 8 | -37.8467, 145.1135 |
| Myrtaceae | *Callistemon viminalis* | Creek bottlebrush "Little John" cultivar | 16 | 4 | 16 | -37.8296, 145.0216 |
| Myrtaceae | *Callistemon viminalis* | Creek bottlebrush "Little John" cultivar | 16 | 2 | 8 | -37.9091, 145.1326 |
| Myrtaceae | *Callistemon viminalis* | Creek bottlebrush "Little John" cultivar | 16 | 2 | 16 | -38.1302, 145.2705 |
| Myrtaceae | *Leptospermum laevigatum* | Coastal tea-tree | 32 | 8 | 32 | -37.8296, 145.0217 |
| Myrtaceae | *Leptospermum laevigatum* | Coastal tea-tree | 16 | 8 | 16 | -37.723, 145.0481 |
| Myrtaceae | *Leptospermum laevigatum* | Coastal tea-tree | 32 | 16 | 32 | -37.9124, 145.1404 |
| Myrtaceae | *Leptospermum laevigatum* | Coastal tea-tree | 16 | 8 | 32 | -38.0054, 145.0818 |
| Myrtaceae | *Leptospermum laevigatum* | Coastal tea-tree | 16 | 8 | 32 | -37.8303, 144.9767 |
| Myrtaceae | *Leptospermum lanigerum* | Woolly tea-tree | 16 | 8 | 16 | -37.6896, 144.5931 |
| Myrtaceae | *Leptospermum lanigerum* | Woolly tea-tree | 16 | 8 | 16 | -37.8401, 145.1094 |
| Myrtaceae | *Leptospermum lanigerum* | Woolly tea-tree | 16 | 8 | 16 | -37.716, 145.0494 |
| Myrtaceae | *Leptospermum lanigerum* | Woolly tea-tree | 8 | 4 | 8 | -37.9077, 145.1372 |
| Myrtaceae | *Leptospermum lanigerum* | Woolly tea-tree | 8 | 4 | 16 | -38.1299, 145.2669 |
| Myrtaceae | *Melaleuca ericifolia* | Swamp paperbark | 16 | 4 | 16 | -37.9077, 145.1375 |
| Myrtaceae | *Melaleuca ericifolia* | Swamp paperbark | 32 | 16 | 16 | -37.8466145.1112 |
| Myrtaceae | *Melaleuca ericifolia* | Swamp paperbark | 16 | 8 | 32 | -37.7156145.0491 |
| Myrtaceae | *Melaleuca ericifolia* | Swamp paperbark | 16 | 4 | 16 | -38.1299145.2669 |
| Myrtaceae | *Melaleuca ericifolia* | Swamp paperbark | 16 | 8 | 32 | -37.8282144.98 |
| Myrtaceae | *Melaleuca fulgens* | Scarlet Honeymyrtle | 8 | 8 | 8 | -37.8098145.0912 |
| Myrtaceae | *Melaleuca fulgens* | Scarlet Honeymyrtle | 8 | 8 | 16 | -37.8298145.0219 |
| Myrtaceae | *Melaleuca fulgens* | Scarlet Honeymyrtle | 16 | 4 | 8 | -37.9075145.1314 |
| Myrtaceae | *Melaleuca fulgens* | Scarlet Honeymyrtle | 8 | 4 | 16 | -37.8164145.3802 |
| Myrtaceae | *Melaleuca fulgens* | Scarlet Honeymyrtle | 8 | 8 | 8 | -38.1304145.2701 |
| Myrtaceae | *Melaleuca hypericifolia* | Hillock bush | 8 | 4 | 16 | -37.8099145.091 |
| Myrtaceae | *Melaleuca hypericifolia* | Hillock bush | 16 | 8 | 32 | -37.79145.0388 |
| Myrtaceae | *Melaleuca hypericifolia* | Hillock bush | 8 | 8 | 16 | -37.8298145.0219 |
| Myrtaceae | *Melaleuca hypericifolia* | Hillock bush | 16 | 16 | 32 | -37.816145.3809 |
| Myrtaceae | *Melaleuca hypericifolia* | Hillock bush | 16 | 8 | 32 | -37.8295144.9824 |
| Myrtaceae | *Melaleuca lanceolata* | Moonah | 64 | 32 | 32 | -37.9094145.1398 |
| Myrtaceae | *Melaleuca lanceolata* | Moonah | 32 | 32 | 64 | -37.81145.0912 |
| Myrtaceae | *Melaleuca lanceolata* | Moonah | 64 | 16 | 64 | -37.7161145.0496 |
| Myrtaceae | *Melaleuca lanceolata* | Moonah | 32 | 16 | 32 | -38.1268145.2705 |
| Myrtaceae | *Melaleuca lanceolata* | Moonah | 32 | 32 | 32 | -37.8325144.9815 |
| Myrtaceae | *Melaleuca linariifolia* | Narrow-leaved paperbark | 32 | 8 | 16 | -37.9089145.1388 |
| Myrtaceae | *Melaleuca linariifolia* | Narrow-leaved paperbark | 16 | 16 | 16 | -37.8096145.091 |
| Myrtaceae | *Melaleuca linariifolia* | Narrow-leaved paperbark | 16 | 8 | 16 | -37.8389145.0721 |
| Myrtaceae | *Melaleuca linariifolia* | Narrow-leaved paperbark | 8 | 8 | 8 | -38.1283145.2713 |
| Myrtaceae | *Melaleuca linariifolia* | Narrow-leaved paperbark | 16 | 16 | 16 | -37.8333144.9831 |

MIC defines the minimum inhibitory concentration (mg/mL) of methanolic leaf extracts derived from each test plant against selected test microorganisms

**Table S4:** Minimum inhibitory concentration and location data for all replicates of “putatively non-antimicrobial” test plants

| Family | Scientific name | Common name | *Salmonella* ser. Typhimurium MIC (mg/mL) | *E. faecalis* MIC (mg/mL) | *E. coli* MIC (mg/mL) | Collection location (GPS coordinates) |
| --- | --- | --- | --- | --- | --- | --- |
| Pittosporaceae | *Bursaria spinosa ssp. spinosa* | Sweet bursaria | 64 | 16 | 32 | -37.691, 144.5899 |
| Pittosporaceae | *Bursaria spinosa ssp. spinosa* | Sweet bursaria | 32 | 16 | 64 | -37.841, 145.1103 |
| Pittosporaceae | *Bursaria spinosa ssp. spinosa* | Sweet bursaria | 32 | 8 | 32 | -37.7161, 145.0496 |
| Pittosporaceae | *Bursaria spinosa ssp. spinosa* | Sweet bursaria | 32 | 8 | 64 | -37.9088, 145.1312 |
| Pittosporaceae | *Bursaria spinosa ssp. spinosa* | Sweet bursaria | 32 | 16 | 32 | -37.8158, 145.3808 |
| Cyperaceae | *Carex appressa* | Tall sedge | 64 | 64 | 64 | -37.9088, 145.1411 |
| Cyperaceae | *Carex appressa* | Tall sedge | 32 | 64 | 64 | -37.9088, 145.1411 |
| Cyperaceae | *Carex appressa* | Tall sedge | 32 | 64 | 32 | -37.9088, 145.1411 |
| Cyperaceae | *Carex appressa* | Tall sedge | 64 | 32 | 64 | -37.8315, 144.9776 |
| Cyperaceae | *Carex appressa* | Tall sedge | 32 | 32 | 64 | -37.9088, 145.1411 |
| Goodeniaceae | *Goodenia ovata* | Hop Goodenia | 64 | 16 | 64 | -37.8099, 145.0908 |
| Goodeniaceae | *Goodenia ovata* | Hop Goodenia | 64 | 16 | 32 | -37.7962, 145.0013 |
| Goodeniaceae | *Goodenia ovata* | Hop Goodenia | 64 | 16 | 64 | -37.6895, 144.593 |
| Goodeniaceae | *Goodenia ovata* | Hop Goodenia | 64 | 32 | 32 | -37.8298, 145.0214 |
| Goodeniaceae | *Goodenia ovata* | Hop Goodenia | 32 | 32 | 32 | -37.7159, 145.0494 |
| Goodeniaceae | *Goodenia ovata* | Hop Goodenia | 32 | 16 | 32 | -37.8161, 145.3809 |
| Malvaceae | *Gynatrix pulchella* | Hemp bush | 32 | >64 | 32 | -37.7969, 145.0044 |
| Malvaceae | *Gynatrix pulchella* | Hemp bush | 32 | >64 | 32 | -37.8459, 145.1121 |
| Malvaceae | *Gynatrix pulchella* | Hemp bush | 64 | 64 | 32 | -37.8298, 145.0215 |
| Malvaceae | *Gynatrix pulchella* | Hemp bush | 64 | >64 | 32 | -37.7162, 145.0495 |
| Malvaceae | *Gynatrix pulchella* | Hemp bush | 64 | >64 | 64 | -37.9074, 145.1371 |
| Rutaceae | *Philotheca myoporoides* | Long-leaf Waxflower | 32 | 64 | 64 | -37.8111, 145.0907 |
| Rutaceae | *Philotheca myoporoides* | Long-leaf Waxflower | 32 | >64 | 32 | -37.8296, 145.0215 |
| Rutaceae | *Philotheca myoporoides* | Long-leaf Waxflower | 32 | >64 | 32 | -37.8612, 145.0408 |
| Rutaceae | *Philotheca myoporoides* | Long-leaf Waxflower | 32 | 64 | 32 | -37.8733, 145.0319 |
| Rutaceae | *Philotheca myoporoides* | Long-leaf Waxflower | 64 | >64 | 64 | -37.8167, 145.3803 |
| Prostantheroideae | *Westringia fruticosa* | Coastal rosemary | 32 | 64 | 32 | -37.8111, 145.0907 |
| Prostantheroideae | *Westringia fruticosa* | "Jervis Gem" cultivar | 16 | 64 | 32 | -37.8281, 145.0217 |
| Prostantheroideae | *Westringia fruticosa* | "Blue Gem" cultivar | 16 | 32 | 64 | -37.8731, 145.0307 |
| Prostantheroideae | *Westringia fruticosa* | "Grey box" cultivar | 32 | 64 | 32 | -37.9099, 145.1387 |
| Prostantheroideae | *Westringia fruticosa* | "Grey box" cultivar | 32 | 32 | 32 | -37.8163, 145.3801 |

MIC defines the minimum inhibitory concentration (mg/mL) of methanolic leaf extracts derived from each test plant against selected test microorganisms

**Table S5:** Significant comparisons in antimicrobial activity between plant species

| Comparison | Kruskal wallis p-value (Dunn's corrected) | Mann Whitney p-value (Bonferroni corrected) |
| --- | --- | --- |
| *Callistemon pallidus vs. Carex appressa* | 0.0023 | < 0.004 |
| *Callistemon pallidus vs. Gynatrix pulchella* | 0.0008 | < 0.004 |
| *Callistemon pallidus vs. Philotheca myoporoides* | 0.0021 | < 0.004 |
| *Callistemon salignus vs. Carex appressa* | 0.0088 | < 0.004 |
| *Callistemon salignus vs. Gynatrix pulchella* | 0.0034 | < 0.004 |
| *Callistemon salignus vs. Philotheca myoporoides* | 0.0081 | < 0.004 |
| *Callistemon sieberi vs. Carex appressa* | 0.0073 | < 0.004 |
| *Callistemon sieberi vs. Gynatrix pulchella* | 0.0028 | < 0.004 |
| *Callistemon sieberi vs. Philotheca myoporoides* | 0.0067 | < 0.004 |
| *Callistemon viminalis vs. Melaleuca lanceolata* | 0.0008 | < 0.004 |
| *Callistemon viminalis vs. Carex appressa* | <0.0001 | < 0.004 |
| *Callistemon viminalis vs. Goodenia ovata* | 0.0006 | < 0.004 |
| *Callistemon viminalis vs. Gynatrix pulchella* | <0.0001 | < 0.004 |
| *Callistemon viminalis vs. Philotheca myoporoides* | <0.0001 | < 0.004 |
| *Callistemon viminalis vs. Westringia fruticosa* | 0.0008 | < 0.004 |
| *Leptospermum laevigatum vs. Carex appressa* | 0.0398 | < 0.004 |
| *Leptospermum laevigatum vs. Gynatrix pulchella* | 0.0165 | < 0.004 |
| *Leptospermum laevigatum vs. Philotheca myoporoides* | 0.0368 | < 0.004 |
| *Leptospermum lanigerum vs. Melaleuca lanceolata* | 0.0016 | < 0.004 |
| *Leptospermum lanigerum vs. Carex appressa* | <0.0001 | < 0.004 |
| *Leptospermum lanigerum vs. Goodenia ovata* | 0.0011 | < 0.004 |
| *Leptospermum lanigerum vs. Gynatrix pulchella* | <0.0001 | < 0.004 |
| *Leptospermum lanigerum vs. Philotheca myoporoides* | <0.0001 | < 0.004 |
| *Leptospermum lanigerum vs. Westringia fruticosa* | 0.0016 | < 0.004 |
| *Melaleuca ericifolia vs. Carex appressa* | 0.0019 | < 0.004 |
| *Melaleuca ericifolia vs. Gynatrix pulchella* | 0.0007 | < 0.004 |
| *Melaleuca ericifolia vs. Philotheca myoporoides* | 0.0017 | < 0.004 |
| *Melaleuca fulgens vs. Melaleuca lanceolata* | <0.0001 | < 0.004 |
| *Melaleuca fulgens vs. Bursaria spinosa ssp. spinosa* | 0.0103 | < 0.004 |
| *Melaleuca fulgens vs. Carex appressa* | <0.0001 | < 0.004 |
| *Melaleuca fulgens vs. Goodenia ovata* | <0.0001 | < 0.004 |
| *Melaleuca fulgens vs. Gynatrix pulchella* | <0.0001 | < 0.004 |
| *Melaleuca fulgens vs. Philotheca myoporoides* | <0.0001 | < 0.004 |
| *Melaleuca fulgens vs. Westringia fruticosa* | <0.0001 | < 0.004 |
| *Melaleuca hypericifolia vs. Carex appressa* | 0.0007 | < 0.004 |
| *Melaleuca hypericifolia vs. Gynatrix pulchella* | 0.0002 | < 0.004 |
| *Melaleuca hypericifolia vs. Philotheca myoporoides* | 0.0006 | < 0.004 |
| *Melaleuca linariifolia vs. Carex appressa* | 0.0006 | < 0.004 |
| *Melaleuca linariifolia vs. Gynatrix pulchella* | 0.0002 | < 0.004 |
| *Melaleuca linariifolia vs. Philotheca myoporoides* | 0.0006 | < 0.004 |

Confirmatory post hoc Bonferroni-corrected Mann-Whitney (PBMW) comparisons were performed for all comparisons where Dunn's corrected Kruskal-Wallis P-values were < 0.05. All PBMW p-values for the above comparisons were < 0.004.

# References

1. Read J, Fletcher TD, Wevill T, Deletic A. Plant traits that enhance pollutant removal from stormwater in biofiltration systems. Int J Phytoremediation. 2010;12(1):34-53.

2. Read J, Wevill T, Fletcher T, Deletic A. Variation among plant species in pollutant removal from stormwater in biofiltration systems. Water Res. 2008;42(4):893-902.

3. FAWB. Adoption Guidelines for Stormwater Biofiltration Systems. Melbourne, Australia: Facility for Advancing Water Biofiltration, Monash University, 2009.

4. Payne EGI, Hatt BE, Deletic A, Dobbie MF, McCarthy DT, Chandrasena GI. Adoption Guidelines for Stormwater Biofiltration Systems. Melbourne, Australia: Cooperative Research Centre for Water Sensitive Cities; 2015.

5. Chandrasena G, Pham T, Payne E, Deletic A, McCarthy D. E. coli removal in laboratory scale stormwater biofilters: Influence of vegetation and submerged zone. J Hydrol. 2014;519:814-22.

6. Li Y, McCarthy DT, Deletic A. Escherichia coli removal in copper-zeolite-integrated stormwater biofilters: Effect of vegetation, operational time, intermittent drying weather. Ecol Eng. 2016;90:234-43.

7. Payne EGI, Pham T, Cook PLM, Fletcher TD, Hatt BE, Deletic A. Biofilter design for effective nitrogen removal from stormwater–influence of plant species, inflow hydrology and use of a saturated zone. Wat Sci Tec. 2014;69(6):1312-9.

8. Savoia D. Plant-derived antimicrobial compounds: alternatives to antibiotics. Fut Microbiol. 2012;7(8):979-90.

9. Mithöfer A, Boland W. Plant defense against herbivores: chemical aspects. Annu Rev Plant Biol. 2012;63:431-50.

10. Freeman BC, Beattie GA. An overview of plant defenses against pathogens and herbivores. The Plant Health Instructor. 2008.

11. War AR, Paulraj MG, Ahmad T, Buhroo AA, Hussain B, Ignacimuthu S, et al. Mechanisms of plant defense against insect herbivores. Plant Signal Behav. 2012;7(10):1306-20.

12. Massad TJ, Fincher RM, Smilanich AM, Dyer L. A quantitative evaluation of major plant defense hypotheses, nature versus nurture, and chemistry versus ants. Arthropod-Plant Interactions. 2011;5(2):125-39.

13. Hengstum T, Hooftman DA, Oostermeijer JGB, Tienderen PH. Impact of plant invasions on local arthropod communities: a meta‐analysis. J Ecol. 2014;102(1):4-11.

14. Danjon F, Stokes A, Bakker MR. Root systems of woody plants. In: Eshel A, Beeckman T, editors. Plant Roots: The Hidden Half. 4 ed. Boca Raton, Florida: CRC Press; 2013. p. 29.1–.1.

15. Payne EGI. The Influence of Plant Species and Water Dynamics on Nitrogen Removal Within Stormwater Biofilters [Thesis for the degree of Doctor of Philosophy, Civil Engineering]. Melbourne, Australia: Monash University; 2013.

16. State Flora. Plants tolerant of waterlogging. N.S.W, Australia: Department of Environment, Water and Natural Resources, 2013.

17. APS. Flora of Melbourne. Bull M, Stolfo G, editor. Melbourne, Australia: Australian Plants Society Maroondah Inc. and Hyland House; 2014.

18. Costermans L. Native Trees and Shrubs of South-Eastern Australia. Costermans L, editor. Sydney: Reed New Holland; 2009.

19. Chandrasena G, Pham T, Payne E, Deletic A, McCarthy D. E. coli removal in laboratory scale stormwater biofilters: Influence of vegetation and submerged zone. J Hydrol. 2014;519:814-22.

20. Le Coustumer S, Fletcher TD, Deletic A, Barraud S, Poelsma P. The influence of design parameters on clogging of stormwater biofilters: a large-scale column study. Water Res. 2012;46(20):6743-52.

21. Payne EG, Pham T, Cook PL, Fletcher TD, Hatt BE, Deletic A. Biofilter design for effective nitrogen removal from stormwater–influence of plant species, inflow hydrology and use of a saturated zone. Wat Sci Tec. 2014;69(6):1312-9.

22. Feng W, Hatt BE, McCarthy DT, Fletcher TD, Deletic A. Biofilters for stormwater harvesting: understanding the treatment performance of key metals that pose a risk for water use. Env Sci Tec. 2012;46(9):5100-8.
